# Supplementary material for: Humoral Immunity in Immunosuppressed IBD Patients after the Third SARS-CoV-2 Vaccination: A Comparison with Healthy Control Subjects
Source: Vaccines (Basel). 2023 Aug 24;11(9):1411. doi: 10.3390/vaccines11091411 (PMC10536352; doi:10.3390/vaccines11091411)
Supplement: Supplementary file 1 [file vaccines-11-01411-s001.zip › vaccines-2548390-supplementary.pdf]

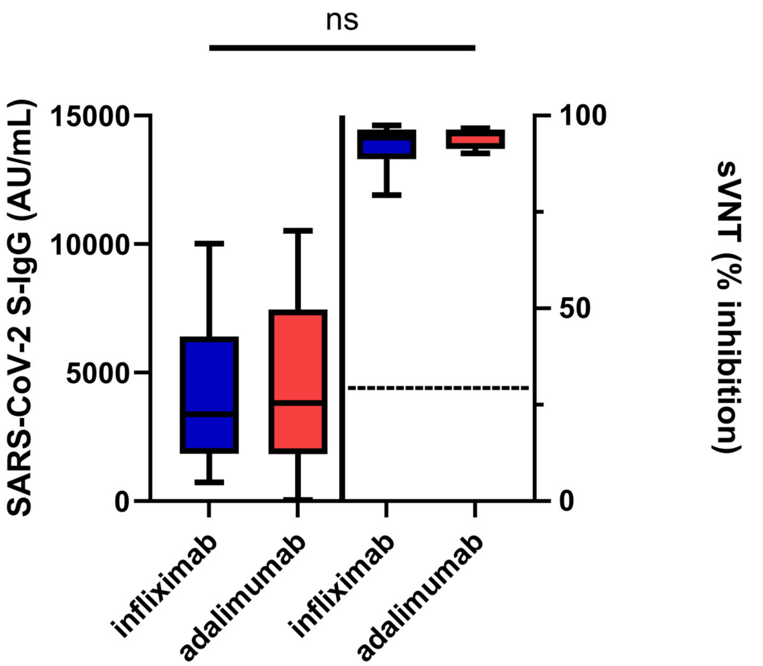

Supplemental Figure S1:

SARS-CoV-2 S-IgG and sVNT levels in IBD patients without prior COVID-19 infection under anti-TNF therapy with adalimumab (n=12) and infliximab (n=24). Ns = not significant;

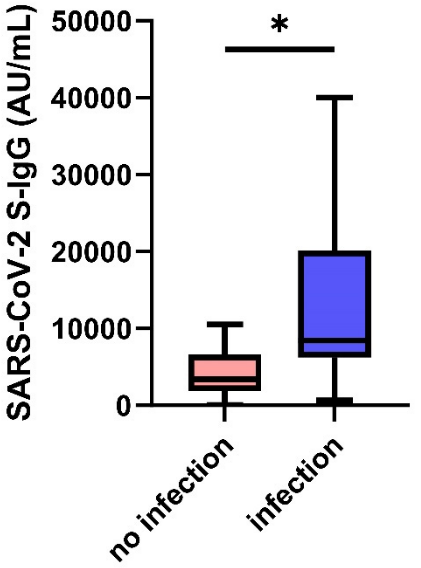

Supplemental Figure S2:

Comparison of IBD patients under anti-TNF therapy after the third COVID-19 vaccination without prior SARS-CoV-2 infection (no infection) with patients after previous SARS-CoV-2 infection (with infection). * $p < 0.05$.

Supplemental Table S1.

**Publication**	**Overall Cohort and Subgroups**	**Vaccination**	**Intervall between third vaccination and blood sampling**	**Results**
**Schell et al. (22)**	n=189 (Anti-TNF, steroids, Anti-TNF/thiopurin-combination, vedolizumab, ustekinumab, tofacitinib, without medication	mRNA vaccines (homologous schedule)	In median 37 days	Reduced anti-S-IgG levels in Patients on Anti-TNF therapy compared to patients without anti-TNF
**Long et al. (23)**	n=408 (steroids, Anti-TNF, Anti-TNF/thiopurine-combination, thiopurine, methotrexate, vedolizumab, ustekinumab, tofacitinib, without therapie)	mRNA vaccines, vector vaccines (homologous and heterologous schedule)	In median 48 days	Reduced anti-S-IgG levels in Patients on Anti-TNF therapy compared to patients without anti-TNF; significant increase of levels after 3 vaccinations compared to 2 vaccinations
**Alexander et al. (25)**	n=352 (Thiopurine, Anti-TNF, anti-TNF/Thiopurin combination, ustekinumab, vedolizumab, tofacitinib, healthy controls)	mRNA vaccines, vector vaccines (homologous and heterologous schedule)	28-49 days	Reduced anti-S-IgG levels in Patients on Anti-TNF therapy compared to patients without anti-TNF; significant increase of levels after 3 vaccinations compared to 2 vaccinations
**Kennedy et al. (26)**	Anti-TNF (n=918), vedolizumab (n=442)	mRNA vaccines (homologous schedule)	In median 5 weeks	Reduced anti-S-IgG levels in Patients on Anti-TNF therapy compared to patients without anti-TNF; significant increase of levels after 3 vaccinations compared to 2 vaccinations; significant more breakthrough infections in patients on anti-TNF therapy
**Liu et al. (27)**	Anti-TNF (n=871), vedolizumab (n=417)	mRNA vaccines, vector vaccines (homologous and heterologous schedule)	In median 40 days	Reduced anti-S-IgG levels in Patients on Anti-TNF therapy compared to patients without anti-TNF; significant increase of levels after 3 vaccinations compared to 2 vaccinations; significant more breakthrough infections in patients on anti-TNF therapy
